# Discovery of symbiotic nitrogen fixation and chemoautotrophy in cold-water corals

**DOI:** 10.1038/srep17962

**Published:** 2015-12-08

**Authors:** Jack J. Middelburg, Christina E. Mueller, Bart Veuger, Ann I. Larsson, Armin Form, Dick van Oevelen

**Affiliations:** 1 Department of Earth Sciences, Utrecht University, P.O. Box 80.021, 3508 TA Utrecht, The Netherlands; 2 Department of Ecosystem Studies, Royal Netherlands Institute for Sea Research (NIOZ-Yerseke), P.O. Box 140, 4400 AC Yerseke, The Netherlands; 3 Dept. of Marine Sciences, Tjärnö, University of Gothenburg, 452 96 Strömstad, Sweden; 4 GEOMAR, Helmholtz Centre for Ocean Research, Düsternbrooker Weg 20, 24105 Kiel, Germany

## Abstract

Cold-water corals (CWC) are widely distributed around the world forming extensive reefs at par with tropical coral reefs. They are hotspots of biodiversity and organic matter processing in the world’s deep oceans. Living in the dark they lack photosynthetic symbionts and are therefore considered to depend entirely on the limited flux of organic resources from the surface ocean. While symbiotic relations in tropical corals are known to be key to their survival in oligotrophic conditions, the full metabolic capacity of CWC has yet to be revealed. Here we report isotope tracer evidence for efficient nitrogen recycling, including nitrogen assimilation, regeneration, nitrification and denitrification. Moreover, we also discovered chemoautotrophy and nitrogen fixation in CWC and transfer of fixed nitrogen and inorganic carbon into bulk coral tissue and tissue compounds (fatty acids and amino acids). This unrecognized yet versatile metabolic machinery of CWC conserves precious limiting resources and provides access to new nitrogen and organic carbon resources that may be essential for CWC to survive in the resource-depleted dark ocean.

Microbes involved in nitrogen transformations occur in symbiosis with a wide range of marine eukaryotes, including shipworms, diatoms, sponges and tropical corals^1^,^2^. Symbiotic relationships in tropical corals include not only interactions between the coral and photoautotrophic dinoflagellates (zooxantellae), but also involves cyanobacteria that reduce dinitrogen gas (N_2_) to ammonia that subsequently can be used by the dinoflagellate-coral association^2^,^3^,^4^ or oxidized to nitrate by nitrifying symbiotic microbes^5^. This intense nitrogen cycling in tropical corals is key to understanding their functioning^2^ and enables them to survive in the oligotrophic tropical seas^6^,^7^.

Cold-water corals are ecosystem engineers distributed at water depths more than 50 m across the globe^8^,^9^ and provide a habitat for >2700 species^10^. They live in the dark ocean and are therefore thought to depend on the arrival of organic matter produced in the distant sunlit surface ocean^11^,^12^. This organic matter rapidly degrades during the downward transit rendering a low availability of energy and organic nutrients in the deep sea. CWC have therefore adopted opportunistic feeding strategies utilizing various organic resources and preferentially retaining nitrogen^13^,^14^. CWCs are hotspot of organic matter processing relative to bare sediments and therefore contribute disproportionally to oxygen consumption and dissolved inorganic carbon and nutrient releases^11^,^12^,^15^. Khripounoff *et al.*^15^ reported high coral ammonium excretion rates while the ammonium concentration in the surrounding water was low, implying a high ammonium turnover. Maier *et al.*^16^,^17^ studied dissolved inorganic nutrient release by CWC and found consistent release of phosphate and ammonium and sometimes release of nitrite and nitrate as well. Ambient water concentrations were as low at ~1 *μ*mol L^−1^, as found also for other Atlantic cold-water coral reefs^18^, suggesting high nutrient turnover rates, while the release of nitrate and nitrite hints at nitrification activity by the CWC holobiont, i.e. the coral and its associated microbes.

These apparent conflicting observations can be reconciled if CWC retain nitrogen by efficient recycling or have access to new nitrogen sources that compensates for losses. Here we use ^15^N-labeled dinitrogen, nitrate and ammonium to elucidate nitrogen transformation pathways by the cold-water coral *Lophelia pertusa*, a holobiont that is dominant in the North Atlantic^9^,^13^. Moreover, we use ^13^C-labelled bicarbonate to investigate whether inorganic carbon was fixed by chemoautotrophs and transferred into coral tissue and different tissue components including hydrolysable amino acids (HAAs), total fatty acids (TFAs) and phospholipid-derived fatty acids (PLFAs). Compound specific isotope analysis of bacterial PLFAs allowed us to trace the flow of carbon from the dissolved inorganic carbon pool via chemoautotrophic bacteria to the CWC.

## Results

### Nitrogen cycling

**Figure 1:**
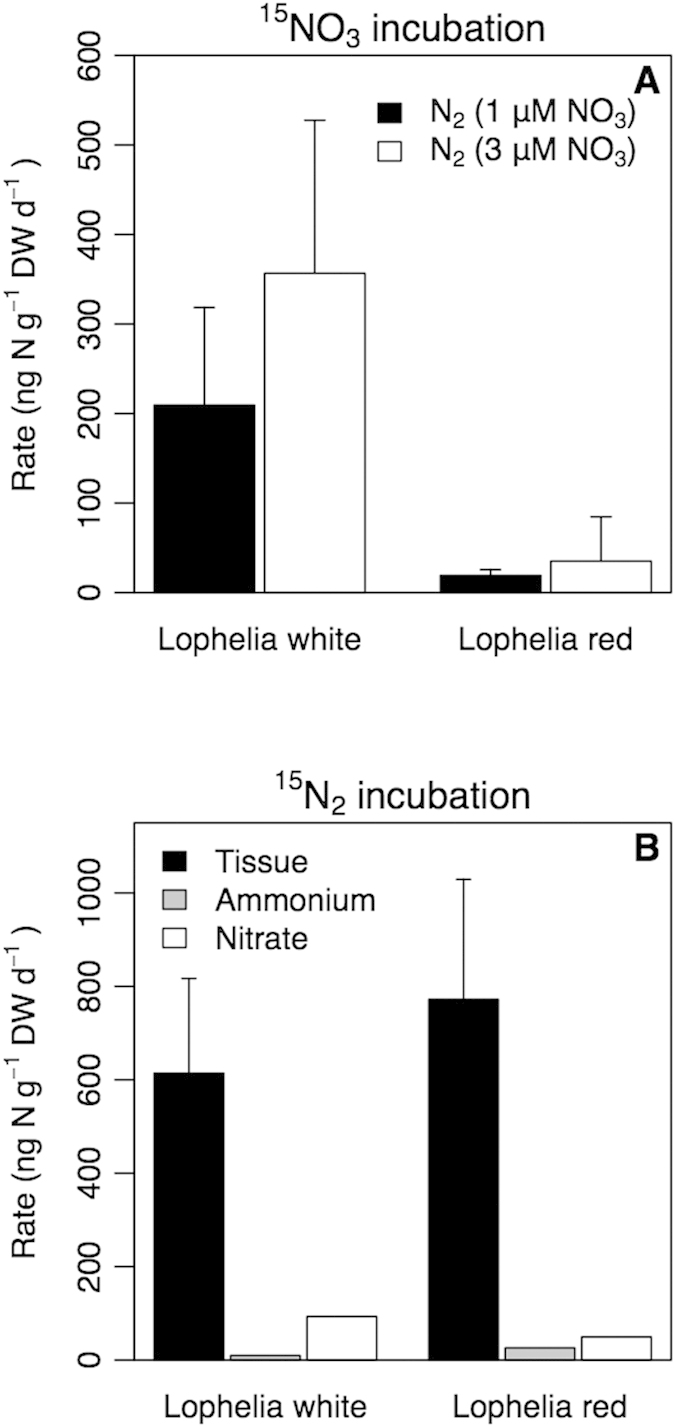
(**A**)^15^NO_3_^−^ addition experiment. Denitrification associated with white and red *L. pertusa* from the Trondheim Fjord. Tracer was added at two concentration levels (see M&M). (**B**) ^15^N_2_ addition experiment. Fixation of N_2_ based on tracer incorporation in coral tissue of red and white *L. pertusa* (Trondheim Fjord) and transfer to dissolved NH_4_^+^ and NO_3_^−^ pools. Results are shown as average ± SD.

Both the white and red phenotypes of *L. pertusa* from Trondheim fjord (Norwegian Shelf) were studied for nitrogen transformation activities using ^15^N labeled substrates. Addition of ^15^NO_3_ resulted in the formation of ^15^N-labelled nitrogen gas within 24 hours (Fig. 1A). Denitrification rates in white *L. pertusa* were significantly higher than those in the red phenotype at low nitrate (Kruskal-Wallis, p < 0.05).

Within 24 hours, ^15^N added in the form of N_2_ was traced into the organic coral tissue of both color morphs (Fig. 1B), indicating that the holobiont *L. pertusa* performs N_2_ fixation, an energy-demanding process, in the dark ocean. Moreover, nitrogen fixation was higher than denitrification for both phenotypes indicating that CWC are a source of fixed nitrogen to the deep ocean. Within 24 hours, net fixation was about 85–90% of gross nitrogen fixation. About 6–13% of the ^15^N_2_ fixed by nitrogen-fixing symbionts was excreted directly as ammonium by the symbionts or regenerated to ammonium and subsequently nitrified (Fig. 1B) indicating a tight coupling between ammonium release and consumption. Gross production and consumption of ammonium based on isotope dilution calculations^19^ varied between 2.4 and 6.9 *μ*g N g^−1^ DW d^−1^ and 0.7 and 1.2 *μ*g N g^−1^ DW d^−1^, respectively (Table 1) and were much higher than rates of nitrification, denitrification and nitrogen fixation. Nitrification contributed less than 1% to ammonium consumption indicating most ammonium was assimilated into organic compounds.

### Carbon fixation and ammonium assimilation

White and red *L. pertusa* from Trondheim fjord were exposed to ^15^N-labelled ammonium and ^13^C-labelled dissolved inorganic carbon for 4 to 10 days. Both^15^N and ^13^C were incorporated into coral tissue (Table 2), with no significant differences between color morphs for either ammonium or inorganic carbon fixation. Inorganic carbon fixation by CWC represents the first evidence for chemoautotrophy and we therefore executed a similar experiment with white *L. pertusa* from Tisler reef (Norwegian Skagerrak). Tisler reef *L. pertusa* also assimilated ammonium and inorganic carbon in its tissue and with similar rates (Kruskal-Wallis, p > 0.05; Table 2). The added ^13^C was also incorporated into coral skeleton with rates of 23 ± 16 *μ*g C g^−1^ DW d^−1^ and 33 ± 17 *μ*g C g^−1^ DW d^−1^, for white and red *L. pertusa* from Trondheim fjord respectively, while corals from the Tisler reef showed an incorporation rate of 46 ± 25 *μ*g C g^−1^ DW d^−1^.

**Figure 2:**
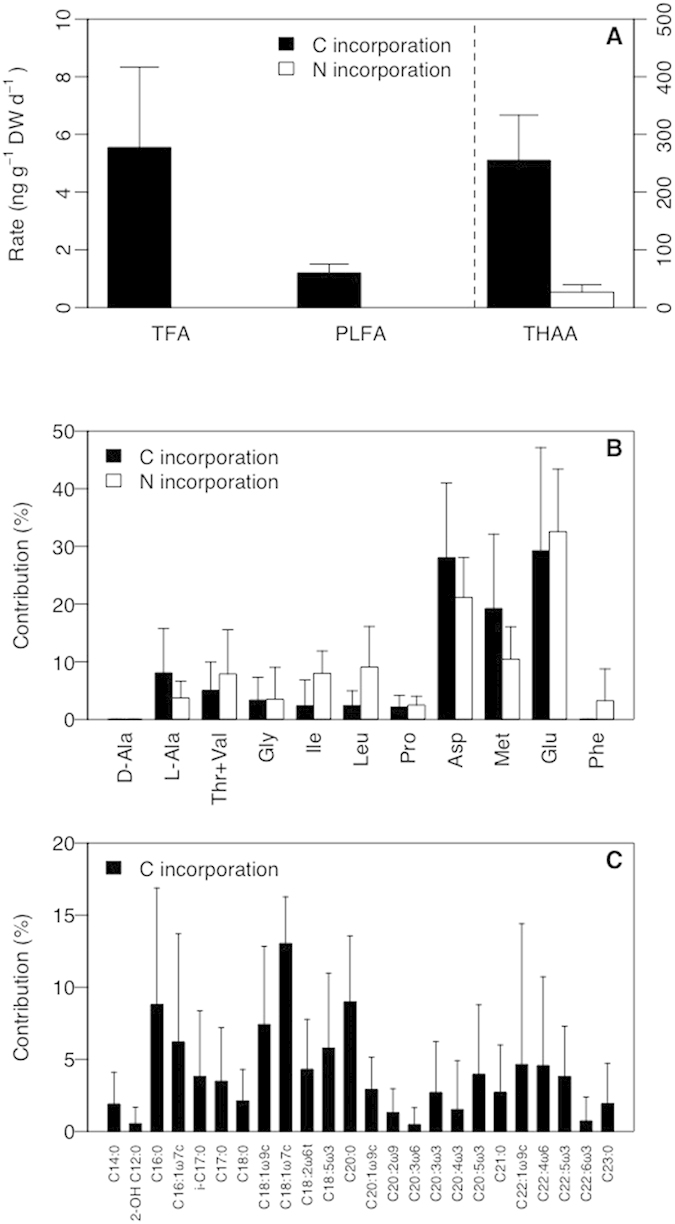
Assimilation of ammonium and fixation of dissolved inorganic carbon into coral tissue and tissue components (total fatty acids (TFA), Polar-lipid derived fatty acids (PLFA) and total hydrolysable amino acids (THAA) of white *L. pertusa* from the Tisler reef. (**A**) Incorporation of dissolved inorganic carbon into TFA, PLFA and THAA pools and assimilation of ammonium into THAA, left axis for TFA and PLFA and right axis for THAA (**B**) incorporation of dissolved inorganic carbon and ammonium into individual amino acids and (**C**) incorporation of dissolved inorganic carbon into individual PLFA. Results are shown as average ± SD.

For the Tisler reef experiment the fate of assimilated inorganic substrates was also traced into specific tissue components (Fig. 2A), including total fatty acids (^13^C), polar-lipid derived fatty acids (^13^C) and amino acids (^13^C, ^15^N). The ^15^N and ^13^C assimilated were incorporated into all amino acids (Fig. 2B), but D-alanine, a bacterial biomarker. Highest tracer recoveries were in glutamine, asparagine and methionine, but also significant amounts were incorporated in essential amino acids such as isoleucine and leucine.

Within the polar-lipid derived fatty acids about a quarter of ^13^C label was incorporated into C16:0, C16:1ω7 and C18:1ω7 (Fig. 2C) which are characteristically dominant in nitrifying and sulfur-oxidizing bacteria^20^. Interestingly, some long-chain PLFAs (C22:1ω9c, C22:4ω6, C22:5ω3 and C20:5ω3) were also readily labeled with ^13^C (Fig. 2C) and thus have been produced *de novo* by the coral since bacteria generally only produce short chain PLFA. This trophic transfer of ^13^C from the bacterial symbionts to the coral host evidently shows that chemoautotrophic bacteria supplement the coral’s carbon and energy demand.

## Discussion

### Nitrogen cycling in CWC

**Figure 3:**
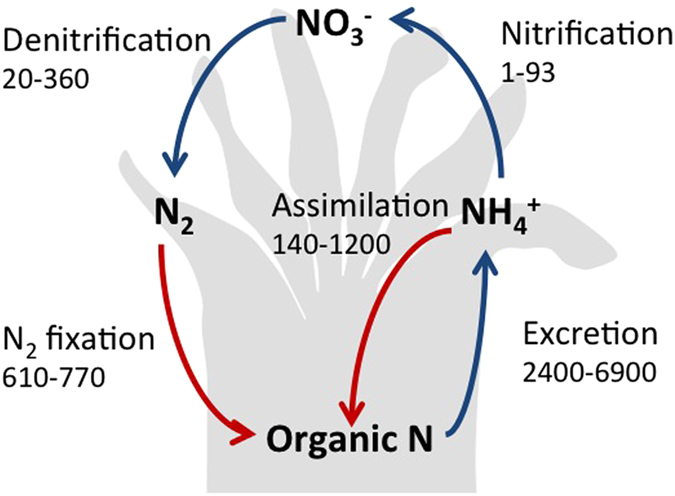
A simplified overview of nitrogen cycling (ng N g^−1^ DW d^−1^) in cold-water corals. Red arrows indicate processes contributing to nitrogen acquisition/retention. Rates are based on those presented in Fig. 1 and Tables 1 and 2.

Living in the resource-depleted dark ocean CWC feed on a variety of organic resources including algae, bacteria, zooplankton, phytodetritus and dissolved organic matter^13^,^14^. This flexibility in heterotrophic feeding enables CWC to optimally acquire the scarce resources. However, high rates of ammonium excretion^15^,^16^,^17^ implicate large nitrogen losses. It is evident that long-term survival also implies conserving limiting resources via efficient recycling. All targeted nitrogen processes were actively mediated by the cold-water coral holobiont *L. pertusa* (Fig. 3). The co-occurrence of ammonium production and assimilation, nitrification, denitrification, and nitrogen fixation indicates a complete nitrogen cycle in cold-water reefs similar to that inferred for tropical reefs^2^,^21^.

The ^15^N_2_ labeling results presented here (Fig. 1B) are consistent with recent studies^22^,^23^ reporting gene sequences of cyanobacteria and the bacterial genus *Vibrio* in *L. pertusa* samples, each of them able to perform the required metabolic pathways for nitrogen fixation^3^,^4^,^24^. These, or related microbes, may be involved in N_2_ fixation by the cold-water coral holobiont. Since photoautotrophy can be excluded in the dark ocean, the microbial symbionts fixing nitrogen in *L. pertusa* are most likely supported by organic compounds released by their coral host to fuel N_2_ fixation^2^,^6^. Respiration by the corals and pelagic microbes may temporarily lower oxygen concentration in the reef water^25^ and thereby favor the activity of the oxygen sensitive N_2_-fixation enzyme nitrogenase^1^,^6^,^24^.

Low oxygen concentrations may also stimulate denitrification, the microbial reduction of nitrate to dinitrogen as observed in our experiments (Fig. 1A). It is unclear when or where denitrification occurs in *L. pertusa*, but the required anaerobic conditions suggests that denitrification can occur during polyp retraction, in micro-niches in the coral mucus layer or in the gut cavity^26^. Denitrification rates were higher in white than red *L. pertusa* (Fig. 1A). This difference is consistent with the dominance of mixotrophic Rodobacteraceae in white *L. pertusa*^22^, a family that includes denitrifiers^27^.

Our experiments provided multiple indications for an actively nitrifying community. First, ^15^N added in the form of ammonium was readily transferred to nitrate (Table 1). Second, 6–13% of the ^15^N_2_ fixed by nitrogen-fixing symbionts was regenerated and subsequently nitrified (Fig. 1B). Nitrification activity in CWC is consistent with the recent documentation of marine group 1 Thaumarchaeota in *L. pertusa* at Rockall Bank^28^, a group of organisms involved in nitrification^29^. Moreover, PLFA biomarkers for nitrifying and sulphur-oxidizing bacteria (C16:0, C16:1ω7 and C18:1ω7) incorporated most of the inorganic ^13^C among the PLFA (Fig. 2C).

This versatility in CWC nitrogen recycling reduces loss of nitrogen, allows for adjustment to changing availability in quantity and quality of resources and may thus be key to survival in the resource-depleted dark ocean. Moreover, nitrogen fixation by microbial symbionts provides corals with new organic nitrogen, complementing organic nitrogen obtained from their diet (Fig. 3). Ammonium production (2400–6900 ng N per gram dw per day) approximates total nitrogen acquisition via heterotroph feeding and nitrogen fixation (610–770 ng N per gram dw per day), indicating that nitrogen fixation contributes between 9 and 32% to CWC nitrogen requirement. This additional N source may explain the release of mucus with a lower than Redfield C:N ratio^25^.

Although this efficient nitrogen recycling and nitrogen fixation may be beneficial for CWC functioning, simple calculations indicate that is of limited importance for the nitrogen cycle of the deep ocean. On the basis of an average nitrogen fixation rate of 667 ng N per gram dw per day (Fig. 1B) or 204 ng N per polyp per day, and ∼12,000 polyps per m^2^ (11), one obtains ∼0.9 g N m^−2^ y^−1^. This is likely an upper estimate because of the high density of polyps in the studied system, but it is similar to nitrogen fixation rates found in coastal sediments^30^,^31^. Assuming a CWC reef extension similar to that of tropical coral reefs^8^, this relates to a global nitrogen fixation rate of ∼0.5 Tg N y^−1^, which is an insignificant contribution to global open ocean nitrogen fixation (∼140 to ∼177 Tg N y^−1^), and only a small contribution to total shelf nitrogen fixation (∼17 Tg N y^−1^ 32).

### Chemoautotrophy in CWC

*L. pertusa* showed significant rates of inorganic carbon fixation in organic tissue (Table 2) and tissue components (HAA, TFA and PLFA; Fig. 2A), indicating a role of chemolithoautotrophs in moderating carbon flow to the coral. The energy for chemoautotrophy comes from the oxidation of substances such as ammonium or reduced sulfur. The observed stoichiometry of 10–100 *μ*mol HCO_3_^−^ fixed for about 1 *μ*mol NO_3_^−^ produced differs considerably from the typical nitrifier stoichiometry of 0.1 μmol HCO_3_^−^ fixed for 1 *μ*mol NO_3_^−^ produced. This stoichiometric mismatch indicates that other chemoautotrophs, such as sulfur oxidizing bacteria of which sequences have been observed in *L. pertusa*^22^, also may have contributed to inorganic carbon fixation. PLFA results indicate that most of the ^13^C label was recovered in C16:0, C16:1ω7 and C18:1ω7. These PLFA are abundant not only in nitrifying, but also sulfur-oxidizing bacteria^20^.

While inorganic carbon was initially fixed by chemoautotrophic symbionts, the carbon was subsequently transferred to the coral as evidenced by the appearance of label in PLFA with a chain length >20, in particular C22:1ω9c, C22:4ω6, C22:5ω3 and C20:5ω3, which must have been produced by the animal. However, whether coral preys on chemoautotrophic bacteria contained within the mucus layer^33^ or whether the bacteria release organic compounds, which the coral then takes up remains to be resolved.

Microbial assimilation of inorganic carbon and ammonium and subsequent transfer to the coral is also evident from the labeling pattern of coral amino acids (Fig. 2B). The ^13^C and ^15^N assimilated was primarily recovered in glutamine and asparagine, consistent with known pathways of ammonium assimilation and amino acid synthesis^34^. Striking are the high enrichment of methionine and formation of isoleucine, leucine, phenylalanine and valine+threonine (lumped because not well resolved in the chromatogram), because these are considered to be essential amino acids, which many animals are considered either incapable of synthesizing or only synthesizing it in insufficient amounts to meet their metabolic needs^34^. These results are in agreement with *de novo* synthesis observed for tropical corals^34^, indicating that tropical and cold-water coral holobionts are able to synthesize putative “essential” amino acids. The *de novo* synthesis of amino acids by CWC poses a challenge to the use of compositional and isotope data in diet studies that are based on putative heterotrophic feeding. These newly formed essential amino acids may be transferred up the reef-associated food web, because of corallivory by echinoids as recently reported on Atlantic reefs^35^.

The intensive recycling of nitrogen, the assimilation of ammonium and inorganic carbon into coral tissue and the *de novo* synthesis of putative essential amino acids and fatty acids (including C20:5ω3 and C22:6ω3) suggest that natural abundance isotope and biomarker approaches should be used with care when based on the concept of putative heterotrophic feeding. Intensive recycling of nitrogen, *in situ* nitrogen fixation and assimilation of ammonium may cause changes in bulk coral tissue δ^15^N values complicating diet and trophic level inferences from natural abundance isotope ratios. Similarly, compound-specific isotope analysis of amino acids is increasingly used to infer diet and trophic transfers, including deep-sea corals^36^,^37^,^38^. The underlying rationale is a division between non-essential and essential amino acids. Our data clearly show that, at least for *L. pertusa*, all amino acids can be generated *de novo*, including the putative essential isoleucine, leucine and threonine+valine. However, comparing inorganic carbon fixation from this study with coral respiration^39^,^40^ it appears that chemoautotrophy provides less than 2% of the energy supply to *L. pertusa*.

Overall, chemoautotrophy, nitrogen fixation and efficient recycling of nitrogen by microbial symbionts may be perquisites for the longevity of CWC in the dark, resources-limited ocean. However, our findings require follow-up studies to elucidate the importance of chemoautotrophy, nitrogen fixation and efficient nitrogen recycling for CWC food web functioning, for carbon and nitrogen budgets and the use of deep-sea corals for paleoenvironmental reconstructions.

## Material and Methods

The experiments were either carried out on board of the RV Poseidon during the cruise P420 to Trondheim fjord (Norway) or in the laboratory at the Sven Lovén Centre for Marine Sciences in Tjärnö, Sweden (Table 3).

### Sampling locations and maintenance

The corals used in this study were harvested from two different locations. Red and white *L. pertusa* were collected at 30–40 m deep in the Trondheim fjord using the manned submersible JAGO during the Poseidon cruise P420 in September 2011. Coral fragments were cut on-board in small pieces (~2–4 g DW (dry weight) piece^−1^ and ~7–11 polyps piece^−1^) to fit the incubation bottles and were acclimated 2 to 3 days in a 500 L tank filled with seawater at a temperature of 7–8 °C. No food was supplied during the acclimation period. Red and white corals from Trondheim fjord were used to measure transfer from the inorganic carbon pool to coral organic tissue and for a detailed nitrogen cycling study involving quantification of N_2_-fixation, nitrification, denitrification and ammonium assimilation/release. Shipboard incubations with CWC from Trondheim fjord for nitrogen transformation activities were done at 7 °C and in the dark, and replicated three times for each treatment and color morph. Besides the shaking provided by the movement of the ship, every 6 to 8 hours each incubation bottle was gently shaken by hand to mix the incubation water.

At the Tisler reef 13, white *L. pertusa* branches were collected specifically for a detailed investigation of the assimilation of ammonium and the fixation of dissolved inorganic carbon and incorporation into coral tissue components (fatty acids, amino acids). The Tisler reef is located at a water depth of 75–155 m at the border between Norway and Sweden and samples were taken at 110 m using the remotely operated vehicle Sperre Subfighter 7500 DC. After transporting the corals in cooling boxes filled with cold seawater (7–8 °C) to the Sven Lovén Centre, samples were clipped to a similar size as those from Trondheim fjord (3.9 ± 4.3 g DW piece^−1^ and 9.3 ± 1.1 polyps piece^−1^). The Tisler corals were maintained in aquaria (10 L) placed in a dark thermo-constant room (7 °C) for 3 months. The aquaria were continuously flushed with sand-filtered (1–2 mm particle size) water from 45 m depth out of the adjacent Koster fjord (salinity 31–34) (~1 l min^−1^). Corals were fed with larvae (nauplii) of the brine shrimp *Artemia spp.* every 3 to 4 days following common procedures at Sven Lovén Centre at Tjärnö^14^,^40^.

### Experimental procedures

Nitrogen transformation processes are often tightly coupled with the product of one process functioning as the substrate for the other process (e.g. ammonium regenerated is partly re-assimilated or nitrified during the incubation period). This complicates the experimental design and the interpretation of data. However, it also means that microbial activities can be identified in more than one treatment and that it provides some information on the importance of such couplings.

### ^15^N_2_ addition experiment

^15^N_2_ enriched seawater was produced prior to the experiment by injecting ^15^N_2_ gas in degassed artificial seawater following the protocol of Mohr *et al.*^41^ to guarantee homogenous labeling of dissolved nitrogen. Red (2.6 ± 0.6 g DW piece^−1^, 8.7 ± 3.1 polyps piece^−1^) and white (2.3 ± 0.1 g DW piece^−1^, 7.3 ± 0.6 polyps piece^−1^) *L. pertusa* pieces were placed separately in gas-tight glass bottles (70 ml) filled without headspace with GF/F filtered seawater. After closing the bottles, 7 ml of the ^15^N_2_ enriched seawater was injected through the rubber septum of the lid (replacing an equal volume of unlabeled water), resulting in an enrichment of 10 atom% ^15^N in the incubation vial. Control corals were incubated without ^15^N_2_ enriched seawater while controls for nutrient and background isotopic values were incubated without corals and with and without label addition. Incubations lasted 24-h so that the coral-associated microbes had enough time to process the N_2_, while at the same time anoxic conditions of the incubation could be avoided. From the experimental setup, oxygen consumption rates (3.6 *μ*mol O_2_ g^−1^ DW d^−1^ at 7 °C)^39^ and oxygen solubility (280 *μ*M at 7 °C) we anticipated a 50% depletion during the incubation. At the end of the incubations coral pieces were removed from the bottles and stored frozen for later analysis of ^15^N in the host and symbiont tissue (net N_2_-assimilation). The water was filtered, pooled per treatment (to obtain enough material for analysis) and stored frozen for analysis of nutrient concentrations and ^15^N enrichment of ammonium and nitrate. The appearance of ^15^N tracer in ammonium and nitrate pools reflects direct excretion of ammonium or regeneration of fixed nitrogen and subsequent nitrification (Fig. 1B). Nitrification following nitrogen fixation reflects heterogeneous oxygen conditions.

### ^15^NO_3_^−^ addition experiment

Red (1.7 ± 0.6 g DW piece^−1^, 3.5 ± 1.3 polyps piece^−1^) and white *L. pertusa* pieces (1.7 ± 0.7 g DW piece^−1^, 3.5 ± 1.3 polyps piece^−1^) were placed in gas tight glass bottles (70 ml) filled with GF/F filtered seawater enriched with two concentrations of ^15^NO_3_^−^ (1 *μ*M, 3 *μ*M), because ambient concentrations and nitrogen transformation rates were not known beforehand. The control treatment (no coral) contained only filtered seawater or filtered seawater enriched with ^15^NO_3_^−^ at two concentrations and was incubated in parallel. A 24-h incubation period was chosen to give the coral enough time to process the ^15^NO_3_^−^ while avoiding anoxic conditions (see N_2_-fixation). Incubations were terminated by injection of HgCl_2_ and bottles were stored upside down for analysis of ^15^N_2_.

### ^5^NH_4_^+^ addition experiment

Red *L. pertusa* (3.7 ± 0.6 g DW piece^−1^, 11.3 ± 1.5 polyps piece^−1^) and white *L. pertusa* (4.4 ± 1.1 g DW piece^−1^, 10.4 ± 2.5 polyps piece^−1^) were incubated in 250 ml glass bottles filled with 200 ml GF/F filtered sea water enriched with ^15^NH_4_^+^ at two different concentrations (1 *μ*M and 3 *μ*M above the 0.5 *μ*M background). The control treatment (without coral) contained either filtered seawater or filtered seawater enriched with ^15^NH_4_^+^ at the two different treatment levels. After incubation for 48-h corals were removed (the larger incubation bottles prevented low oxygen conditions), the water was filtered (GF/F) and frozen for further analysis of nutrients and ^15^N-enrichment of NO_3_^−^ and NH_4_^+^. The appearance of ^15^N in the nitrate pool is due to nitrification. The change in ammonium concentration and isotopic enrichment of NH_4_^+^ were used to quantify ammonium production and consumption using an isotope dilution technique^19^, originally developed for soils and coastal sediments. As with any isotope labeling technique applied to spatial heterogeneous systems, inferred rates may be biased by non-uniform label distributions.

### ^13^C-DIC and ^15^NH_4_^+^ additions

Ammonium assimilation and fixation of dissolved inorganic carbon were measured in CWC from Trondheim fjord during the cruise and from Tisler reef in the laboratory at Tjärnö (Table 3). During the cruise red and white *L. pertusa* samples from Trondheim fjord were placed separately in incubation chambers (4 L) filled with GF/F filtered sea water and maintained at 8 °C in a water bath. A stirrer in the middle of the chamber maintained water circulation. After a 12-hr period to acclimate from the transfer from the 500L maintenance to 4L experimental chamber, ^13^C-DIC and ^15^NH_4_^+^ were added to the water to attain an enrichment of 30 atom% for both ^15^N and ^13^C. The treatment was replicated three times. Control corals were incubated in parallel without label addition for isotopic background measurements. Every 2.5 days, water was completely exchanged but for about 10% to maintain corals submerged, and new label was added. Incubations lasted for 4 to 10 days to determine the time scale of C and N incorporation into tissue. At the end of the incubation, corals were stored at −20 °C, freeze-dried and kept frozen for further analysis.

In the laboratory, white *L. pertusa* samples from Tisler reef were placed in incubation chambers (10 L) in a thermo-stated room at 7 °C. A motor-driven paddle on top of the chamber (2 rpm) maintained water circulation. Prior to the experiment, chambers were filled with 0.2 *μ*m filtered seawater from 45 m depth out of the Koster fjord (salinity of 33, 7 °C). Three coral fragments were randomly selected and placed in a single chamber. After a 12-hr incubation to acclimate from the transfer from the maintenance to experimental chamber, ^13^C-DIC and ^15^NH_4_^+^ were added to the water to attain an 10 atom% enrichment for both substrates. The experiment was duplicated. Control corals were incubated in parallel without label addition for isotopic background measurements. After an incubation time of 4 days, coral samples were frozen at −20 °C, freeze-dried and stored frozen for further analysis.

### Chemical analyses

Concentration and isotopic composition of dissolved N_2_ (^28^N_2,_^29^N_2_, ^30^N_2_) were determined in the headspace of the incubation bottle, after injection of He which replaced 5 ml of sample water and vigorous shaking, using a Thermo Electron Flash EA 1112 analyzer (EA) coupled to a Delta V isotope ratio mass spectrometer (EA-IRMS) as described in^42^.

NH_4_^+^, NO_2_^−^ and NO_3_^−^ concentration in water samples were determined using automated colorimetric techniques (precision NH_4_^+^ ± 2% SD, NO_2_^−^/NO_3_^−^ ± 3% SD). The isotopic composition of NH_4_^+^ and NO_3_^−^ in the sample was determined in two steps^42^. In the first step, MgO was added to the water sample to convert the NH_4_^+^ to NH_3_, which was subsequently trapped on an acidified (H_2_SO_4_) GF/D filter packed between two Teflon filters floating on the sample surface. In the second step, the remaining NO_3_^−^ was converted to NH_4_^+^ by the addition of Devarda’s Alloy, which was then again trapped on an acidified GF/D filter package as in step one. Finally, both filters were measured for their isotopic composition by EA-IRMS.

For isotope analysis of coral tissues, frozen corals were freeze-dried, weighed and homogenized by grinding with a ball mill for 20 seconds (MM 2000, Retsch, Haan, Germany). A subsample (~30 mg) of ground coral material was decalcified by stepwise acidification with 12M HCl until complete carbonate removal. The remaining organic fraction (tissue + organic skeleton matrix) was measured for C and N concentration and isotopic composition by EA-IRMS. The ^13^C incorporation into the skeleton was determined following^43^.

Coral samples from Tisler Reef were also analyzed for tracer incorporation into total fatty acids (TFA), phospholipid-derived fatty acids (PLFA) and hydrolysable amino acids (HAAs). TFAs were extracted from 0.7 g of grounded coral with a modified Bligh and Dyer method. The PLFA fraction of the total fatty acid extract was separated by silica column (Merck Kieselgel 60)^44^. The TFA and PLFA extracts were derivatized by mild alkaline transmethylation to obtain fatty acid methyl esters (FAME). Preparation of methyl esters was carried out following^44^,^45^. Concentration and carbon isotopic composition of individual TFAs and PLFAs were measured on a gas-chromatograph combustion-interface isotope-ratio mass spectrometer (GC-c-IRMS)^45^.

Hydrolyzable amino acids were extracted and analyzed using a modified protocol^46^. Ground coral samples were first decalcified by repeated addition of 12M HCl drops. The remaining material was then hydrolyzed in 6M HCl at 110 °C for 20h and purified by cation exchange chromatography (Dowex 50WX8 resin). HAAs were derivatized with isopropanol and pentafluoropropionic anhydride and analyzed by GC-c-IRMS for individual AAs concentrations and ^13^C and ^15^N enrichment.

### Calculations

The processing rates/ uptake rates of ^15^N or ^13^C are presented as total N or C processed per gram of DW ground coral. The excess ^15^N or ^13^C is calculated from the difference in heavy isotope fraction (F) between sample and background multiplied by the quantity of nitrogen or carbon^45^,^47^: excess = (F_sample_ − F_backgroung_) × (ng of N or C in sample), where F = R/(R + 1) and the isotope ratio R is calculated directly from the measured δ^15^N or δ^13^C. In order to convert the ^15^N and ^13^C processing rates to total rates, they were multiplied by the ^15^N [i.e., ^15^N/(^15^N+^14^N)] or ^13^C [i.e., ^13^C/(^13^C+^12^C)] fraction of the respective substrate at the start of the incubation, but for the ammonium regeneration data. These were estimated from the concentration and isotopic enrichment of NH_4_^+^ using the isotopic dilution model^19^, which allows estimation of gross and net NH_4_^+^ production and consumption rates. For the nitrification rate measurements, the ^15^N enrichment of the total NH_4_^+^ pool was influenced by strong NH_4_^+^ production during the incubations. To compensate for the resulting isotopic dilution, we used the average ^15^N enrichment of the NH_4_^+^ pool during the incubation period that was calculated from the start value (calculated from natural ^14^NH_4_^+^ concentrations and added ^15^NH_4_^+^ addition) and the end value (directly measured in the extracted NH_4_^+^). Nitrification and denitrification rates determined for coral incubations were corrected for incubation water activity by subtracting the rates obtained from the control incubations. All results are reported as average ± SD. Differences were tested with ANOVA or Kruskal-Wallis if criteria for ANOVA were not met.

## Additional Information

**How to cite this article**: Middelburg, J. J. *et al.* Discovery of symbiotic nitrogen fixation and chemoautotrophy in cold-water corals. *Sci. Rep.* **5**, 17962; doi: 10.1038/srep17962 (2015).

## Figures and Tables

**Table 1:** Results from the ^15^NH_4_^+^ addition experiment.

Treatment	Total Production *μ*g N g^−1^ DW d^−1^	Net production *μ*g N g^−1^DW d^−1^	Consumption *μ*g N g^−1^DW d^−1^	Nitrification ng N g^−1^ DW d^−1^
*L. pertusa* white 1 *μ*M	6.9 ± 1.1	5.7 ± 1.1	1.2 ± 0.1	14.6 ± 15.7
*L. pertusa* white 3 *μ*M	5.0 ± 0.2	4.1 ± 0.1	0.9 ± 0.3	5.9 ± 1.4
*L. pertusa* red 1 *μ*M	6.0 ± 2.0	5.2 ± 1.8	0.8 ± 0.5	3.0 ± 4.8
*L. pertusa* red 3 *μ*M	2.4 ± 0.5	2.3 ± 0.3	0.7 ± 0.6	1.4 ± 2.4

Total and net ammonium production, total consumption and nitrification by red and white *L. pertusa* from Trondheim fjord. All data are the average ± SD and were obtained after an addition of 1 or 3 *μ*M NH_4_^+^ and incubation during two days. Note that nitrification rates are expressed in ng rather than *μ*g N.

**Table 2:** Dissolved inorganic carbon and ammonium incorporation in tissue of red and white *L. pertusa.*

CWC	Carbon Fixation ng C g^−1^ DW d^−1^	Ammonium incorporation ng N g^−1^ DW d^−1^
*L. pertusa* white, Tisler	931 ± 294	261 ± 155
*L. pertusa* white, Trondheim	1016 ± 363	139 ± 22
*L. pertusa* red, Trondheim	1394 ± 464	191 ± 59

Results are shown as average ± SD.

**Table 3:** Experimental design and procedures.

Treatment	Measurement	Process	Incubation Period (d)	Trondheim fjord (P420)	Tisler reef (SLCT)
Nitrogen cycling					
^15^N_2_	15N Tissue ^15^NH_4_^+ 15^NO_3_^−^	N_2_-fixation Ammonium regeneration Nitrification	1	*L. pertusa* white, red	
^15^NO_3_^−^	^15^N_2_	Denitrification	1	*L. pertusa* white, red	
^15^NH_4_^+^	^15^NH_4_^+^ & NH_4_^+ 15^NO_3_^−^	Ammonium regeneration & consumption Nitrification	2	*L. pertusa* white, red	
Assimilation					
^15^NH_4_^+^	15N Tissue*	Ammonium assimilation	4–10	*L. pertusa* white, red	L. pertusa white*
^13^C-DIC	13C Tissue*	DIC-fixation	4–10	*L. pertusa* white, red	L. pertusa white*

All experiments were carried out either during the Poseidon cruise P420 or at the Sven Lovén Centre in Tjärnö (SLC-T).

^*^detailed tissue analysis including ^15^N/^13^C HAAs, ^13^C TFAs and ^13^C PLFAs were only conducted on corals harvested at the Tisler reef.
